# Heterologous vaccine immunogenicity, efficacy, and immune correlates of protection of a modified-live virus porcine reproductive and respiratory syndrome virus vaccine

**DOI:** 10.3389/fmicb.2022.977796

**Published:** 2022-09-23

**Authors:** Jessica Proctor, Iman Wolf, David Brodsky, Lizette M. Cortes, Alba Frias-De-Diego, Glen W. Almond, Elisa Crisci, Tatiane Terumi Negrão Watanabe, James M. Hammer, Tobias Käser

**Affiliations:** ^1^Department of Population Health and Pathobiology, College of Veterinary Medicine, North Carolina State University, Raleigh, NC, United States; ^2^Elanco Animal Health, Greenfield, IN, United States

**Keywords:** porcine reproductive and respiratory syndrome virus (PRRSV), correlates of protection (CoP), vaccination, swine, immunity, pig

## Abstract

Although porcine reproductive and respiratory syndrome virus (PRRSV) vaccines have been available in North America for almost 30 years, many vaccines face a significant hurdle: they must provide cross-protection against the highly diverse PRRSV strains. This cross-protection, or heterologous vaccine efficacy, relies greatly on the vaccine’s ability to induce a strong immune response against various strains—heterologous immunogenicity. Thus, this study investigated vaccine efficacy and immunogenicity of a modified live virus (MLV) against four heterologous type 2 PRRSV (PRRSV-2) strains. In this study, 60 pigs were divided into 10 groups. Half were MOCK-vaccinated, and the other half vaccinated with the Prevacent^®^ PRRS MLV vaccine. Four weeks after vaccination, groups were challenged with either MOCK, or four PRRSV-2 strains from three different lineages—NC174 or NADC30 (both lineage 1), VR2332 (lineage 5), or NADC20 (lineage 8). Pre-and post-challenge, lung pathology, viral loads in both nasal swabs and sera, anti-PRRSV IgA/G, neutralizing antibodies, and the PRRSV-2 strain-specific T-cell response were evaluated. At necropsy, the lung samples were collected to assess viral loads, macroscopical and histopathological findings, and IgA levels in bronchoalveolar lavage. Lung lesions were only induced by NC174, NADC20, and NADC30; within these, vaccination resulted in lower gross and microscopic lung lesion scores of the NADC20 and NADC30 strains. All pigs became viremic and vaccinated pigs had decreased viremia upon challenge with NADC20, NADC30, and VR2332. Regarding vaccine immunogenicity, vaccination induced a strong systemic IgG response and boosted the post-challenge serum IgG levels for all strains. Furthermore, vaccination increased the number of animals with neutralizing antibodies against three of the four challenge strains—NADC20, NADC30, and VR2332. The heterologous T-cell response was also improved by vaccination: Not only did vaccination increase the induction of heterologous effector/memory CD4 T cells, but it also improved the heterologous CD4 and CD8 proliferative and/or IFN-γ response against all strains. Importantly, correlation analyses revealed that the (non-PRRSV strain-specific) serum IgG levels and the PRRSV strain-specific CD4 T-cell response were the best immune correlates of protection. Overall, the Prevacent elicited various degrees of efficacy and immunogenicity against four heterologous and phylogenetically distant strains of PRRSV-2.

## Introduction

The porcine reproductive and respiratory syndrome virus (PRRSV) continues to be the most economically important animal pathogen. This virus causes reproductive failure and respiratory disease, significantly contributing to the porcine respiratory disease complex (PRDC) (Lunney et al., 2016). The respiratory diseases alone have caused an approximate 7.4% drop in annual production output, translating to over $664 million lost annually in the US (Valdes-Donoso et al., 2018). Along with its immunosuppressive capacities (Loving et al., 2015), PRRSV’s high mutation rate allows it to evade the host’s immunity provided either by infection or vaccination (Shi et al., 2010; Murtaugh and Genzow, 2011; Geldhof et al., 2012; Rowland and Lunney, 2017; Zhou et al., 2017; Kvisgaard et al., 2021). These mutations led to a plethora of strains: PRRSV can be divided into two species, type-1 or PRRSV-1, mainly found in Europe, and type-2 or PRRSV-2, prevalent in North America (Brinton et al., 2021). Current classification further divides PRRSV-2 into nine lineages with numerous PRRSV strains (Shi et al., 2010). This high diversity leads to a strong challenge for PRRSV vaccines: they need to protect against the various constantly evolving PRRSV strains present in the swine industry. Lineages 1, 5, 8, and 9 belong to the most prevalent PRRSV-2 lineages (Brar et al., 2015). Based on both the high prevalence of these strains and the necessity to provide broad cross-reactivity, the goal of this study was to assess both heterologous vaccine immunogenicity and efficacy against four PRRSV-2 strains belonging to three highly prevalent lineages—NADC30 and NC174 (lineage 1), VR2332 (lineage 5), and NADC20 (lineage 8).

Vaccine immunogenicity analysis included both the humoral and T-cell immune response. Neutralizing antibodies (nAbs) play a critical role in both viral clearance and defense against re-infection (Lopez and Osorio, 2004). However, several studies have noted the postponed induction of nAbs (Lunney et al., 2016) prior to clearance of viremia (Pileri and Mateu, 2016; Butler et al., 2017). These studies emphasized that clearance of PRRSV can occur before the presence of nAbs. Therefore, while developing nAbs are important in the protection against PRRSV, other factors seem to play a relevant role as well. Viremia reduction or even viral clearance in the absence of nAbs is partly explained by the cell-mediated immune response including the T-cell response, such as IFN-γ production by CD4, CD8, and TCR-γδ T cells (Nan et al., 2017; Kick et al., 2019; Chae, 2021). Based on this central role of T cells in the control of PRRSV, this study includes a detailed analysis of the PRRSV-strain specific proliferative and IFN-γ response of CD4, CD8, and TCR-γδ T cells. In particular, responding CD4 T cells were additionally analyzed on their differentiation from CD8α^–^ naïve into CD8α^+^ antigen-experienced memory/effector cells. This differentiation allows the distinction between a primary and a secondary response of these CD4 T cells.

To accomplish the study of that detailed heterologous vaccine immunogenicity and efficacy, 60 pigs were distributed into 10 groups—five immunized with the Prevacent^®^ PRRS MLV vaccine (hereafter designated as “Prevacent,” Elanco, Greenfield, IN, USA) and five MOCK-inoculated. Four weeks post vaccination, pigs were challenged with one of the above-mentioned PRRSV-2 strains or MOCK-inoculated. Viral shedding and viremia as well as the induced immune response were followed for 2 weeks; then, pigs were euthanized to additionally assess viral loads in bronchoalveolar lavage (BAL), the lungs were submitted for gross and histopathology examinations, and weight of the inguinal lymph nodes were measured. The immune response analysis included the humoral and T-cell immune response: the humoral response was studied not only by quantifying mucosal IgA in nasal swabs and BAL, but also by determining the serum IgG and nAb levels; the systemic T-cell response was analyzed in detail including the proliferative and IFN-γ response of CD4, CD8, and TCR-γδ T cells as well as CD4 T-cell differentiation. Furthermore, these immune parameters were investigated for their correlation to the studied vaccine efficacy parameters—lung pathology, viral shedding, and viremia. In at least one of these parameters, Prevacent partially protected against NADC30, VR2332, and NADC20. Prevacent also induced various levels of heterologous immunity: not only did it induce a strong IgA response in the BAL, a strong systemic IgG response, and increased the prevalence of anti-NADC30, -VR2332, and -NADC20 nAbs, but it also promoted (i) the CD4 T-cell differentiation, (ii) the proliferation of CD4, CD8, and TCR-γδ cells, and (iii) a stronger post-challenge IFN-γ response. The correlation analyses between the vaccine efficacy and immunogenicity parameters revealed two important immune correlates of protection—systemic IgG levels and the CD4 T-cell response. However, in contrast to the ELISA used to quantify the systemic IgG levels, the *in vitro* restimulation method followed by multi-color flow cytometry, determines the CD4 response specific to the challenge strain. Conclusively, for the first time, this study identifies the systemic CD4 T-cell response as a strain-specific immune correlate of protection for PRRSV-2. These immune correlates of protection can strongly facilitate vaccine development; and they can be used to predict vaccine efficacy against newly emerging PRRSV-2 strains.

## Materials and methods

### Study design

**FIGURE 1:**
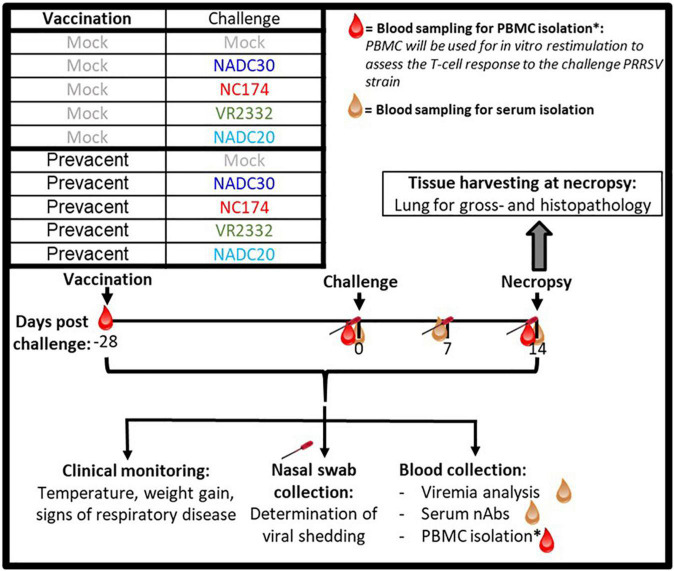
Layout of the PRRSV-2 vaccination and challenge animal trial. Four-week old weaner pigs were distributed into 10 groups—five MOCK– and five Prevacent-vaccinated groups. Pigs were vaccinated at –28 days post challenge (dpc). At day of challenge (0 dpc), each of the five groups received one of five intranasal inoculations—MOCK, NADC30, NC174, VR2332, or NADC20. At 14 dpc, pigs were sacrificed to assess lung pathology. As indicated in the timeline, blood and nasal swabs were collected throughout the study to assess viral loads as well as the humoral and T-cell immune response.

The study design is illustrated in Figure 1: 60 4-week-old weaned pigs from a PRRSV-2-negative farm (NC State University Swine Education Unit, Raleigh, NC, USA) were brought to a BSL-2 Laboratory Animal Research—LAR facility at NC State University, College of Veterinary Medicine (Raleigh, NC, USA). These 60 weaners were randomly divided into ten groups using the GraphPad online tool.^1^ Five groups were intramuscularly (IM) MOCK-inoculated with phosphate-buffered saline (PBS), and five groups with Prevacent as recommended by the manufacturer. Twenty-eight days after vaccination, pigs were intranasally challenged using a Nasal Mist Intranasal Mucosal Atomization Device (Mountainside Medical Equipment, Marcy, NY, USA) (500 μL/nostril; 1 mL total). Each of the MOCK- and MLV-vaccinated (VAC) groups was challenged with a 10^6^ TCID_50/mL_ dose of either NC174 (lineage 1A), NADC30 (lineage 1C), VR2332 (lineage 5), or NADC20 (lineage 8). MOCK-challenged (CHA) pigs were challenged with either 1% bovine serum albumin (BSA) in PBS (3/6 pigs) or Opti-MEM™ (3/6 pigs) as these were the two-suspension media used for the different viral strains. Pigs were clinically monitored daily. At − 28–, 0–, 7–, and 14-days post-challenge (dpc), blood was collected for serum and/or isolation of peripheral blood mononuclear cells (PBMC). Body weight and rectal temperatures were recorded weekly. To facilitate the handling, necropsy was performed over 2 days—15 and 16 dpc. Pigs were euthanized using lethal injection and lungs were collected. First, lungs were assessed and scored by gross examinations and photographs were taken for documentation. Then, lungs were filled with approximately 50 mL PBS, gently massaged, and BAL was harvested for downstream assessment of lung viral loads and the local humoral and cellular immune response. Thereafter, tissue samples were taken for histopathology assessment and scoring, as well as T-cell tissue infiltration. Inguinal lymph nodes were also collected and weighted as clinical indicator of PRRSV exposure (Rossow et al., 1995). The experimental procedures were approved by the NC State University Institutional Animal Care and Use Committee (IACUC) ID# 17-166A (Nov 29, 2017).

### Porcine reproductive and respiratory syndrome virus strains

NC174, NADC20, and NADC30 were provided by Elanco. VR2332 was produced and titrated in house on MA-104 cells. Serum pools from MOCK-VAC, challenged pigs at 7 dpc were sent to ISU VDL for ORF5 sequencing: the sequence analysis confirmed the correct identity of the challenge strains (data not shown, d.n.s).

### Processing of bronchoalveolar lavage, nasal swabs, and blood

Aliquots of 0.4 mL BAL were added to 0.6 mL TriReagent (Ambion, Austin, TX, USA), mixed and stored at −80°C for downstream PRRSV quantification *via* reverse transcription quantitative real-time PCR (qPCR). Remaining BAL was centrifuged at 400 g and 4°C for 10 min to pellet BAL immune cells. Cell pellets were harvested, counted, and used for the analysis of the local T-cell immune response. Supernatants were aliquoted and stored at −80°C for downstream analysis of the humoral immune response *via* IgA ELISA. Nasal swabs were rotated in each nostril and placed in tubes filled with 1 mL PBS. After collection, swabs were vortexed and then rotated in a circular motion pressing against the tube wall before removal of the swabs from from the tube. The PBS from these nasal swabs was aliquoted and stored at −80°C for downstream PRRSV and antibody quantification. Whole blood for serum isolation was collected in SST tubes (BD Bioscience, San Jose, CA, USA) and incubated upright for 30 min. After incubation, blood was spun at 2,000 g for 20 min at 23°C. Serum was harvested and stored in aliquots at −80°C. Whole blood for peripheral blood mononuclear cell (PBMC) isolation was collected in Heparin tubes (BD Bioscience). Isolation of PBMC was performed by density centrifugation using Sepmate tubes (StemCell, Vancouver, BC, Canada) and Ficoll-Paque (GE Healthcare, Uppsala, Sweden). After isolation, PBMCs were used fresh for *in vitro* restimulation to study the PRRSV-strain specific T-cell immune response.

### Viremia and viral loads

Isolated serum, BAL, and nasal swabs were shipped to Iowa State University Veterinary Diagnostic Laboratory (ISU VDL) (Ames, IA, USA) for PRRSV quantification using either a PRRSV-universal or an “Elanco Prevacent-like” specific qPCR. Results were given as Ct values (“Elanco Prevacent-like” qPCR) or genomic copy numbers/mL (universal qPCR).

### Serum anti-PRRSV IgG and anti-PRRSV IgA

Isolated serum and nasal swabs were shipped to ISU VDL. Serum IgG levels were determined with PRRSV X3 enzyme-linked immunosorbent assay (ELISA, IDEXX, Westbrook, ME, USA). PRRSV Oral fluid IgA ELISA was used to determine IgA of nasal swabs.

### Neutralizing antibodies

Serum samples at 0 and 14 dpc were shipped to South Dakota State University Animal Research and Diagnostic Laboratory (SDSU ARDL). Neutralizing antibodies were measured by the fluorescent focus neutralization (FFN) test (Wu et al., 2001). A titer of ≥1:4 was considered positive. Isolated serum was tested against the respective homologous challenge strain. Both MOCK-CHA groups were tested against all four viral strains.

### Macroscopical and histopathology lung examination and scoring, and lymph node weight

At necropsy, lungs and inguinal lymph nodes were collected. Photographs of the dorsal and ventral sides of the lungs were taken. All lung lobes were scored by a swine veterinarian who was blinded to the experimental treatments. For histopathology assessment, lung sections from each of the seven lung lobes were collected—left apical, left cardiac, left diaphragmatic (caudal), right apical, right cardiac, right diaphragmatic (caudal), and intermediate (accessory). Tissue samples were fixed in formaldehyde/Zn fixative (Electron Microscopy Sciences, Hatfield, PA, USA) for 24 h; then, they were transferred to 70% ethanol. The tissue processing, hematoxylin and eosin (H&E) staining, and slide preparation were performed by the NC State University Histology Core facility. Each section was blindly examined by one American College of Veterinary Pathologists board-certified anatomic pathologist as previously described by Halbur et al. (1995). Briefly, the scores were recorded as (0) no microscopic lesions, (1) mild interstitial pneumonia, (2) moderate multifocal interstitial pneumonia, (3) moderate diffuse interstitial pneumonia, or (4) severe interstitial pneumonia. For a general assessment of immune activation, both inguinal lymph nodes were collected at the euthanasia and weighed in grams.

### The porcine reproductive and respiratory syndrome virus challenge-strain specific proliferation of T cells

To measure the proliferation of PRRSV-specific T-cell subsets, freshly isolated PBMCs were stained with CellTrace™ Violet cell Proliferation Kit (Invitrogen) according to the manufacturer’s instructions. Stained cells were seeded in 96-well round-bottom plates (Sarstedt, Nümbrecht, Germany) at 200,000 cells/well. Cells were stimulated for 72 h with medium (MOCK), NC174, NADC20, NADC30, or VR2332 (MOI of 0.1); Concanavalin A (ConA, 2.5 μg/mL, Alfa Aesar) stimulation was used as a positive control. Cells from eight replicates were pooled and stained for flow cytometry analysis according to Table 1. Flow cytometry data were acquired on a Cytoflex using the CytExpert software (Beckman Coulter). Data analysis was performed with FlowJo version 10.5.3 (FLOWJO LLC) with gates based upon relevant FMO controls.

**TABLE 1:** Flow cytometry staining panel.

Antigen	Clone	Isotype	Fluorochrome	Labeling strategy	Primary Ab source	2nd Ab source
CD3	PPT3	IgG1	FITC	Directly conjugated	Southern Biotech	–
CD4	74–12–4	IgG2b	Brilliant Violet 480	Secondary antibody	BEI Resources	Jackson Immunoresearch
CD8α	76–2–11	IgG2a	Brilliant Violet 605	Biotin-Streptavidin	Southern Biotech	Biolegend
TCR-γδ	PGBL22A	IgG1	Alexa Flour 647	Kingfisher	Invitrogen	–
CCR7	3D12	rIgG2a	Brilliant Blue 700	Directly conjugated	BD Biosciences	–
Live/Dead	–	–	Near Infra-red	–	Invitrogen	–
IFN-γ*	P2G10	IgG1	PE	Directly conjugated	BD Biosciences	–
Proliferation^#^	–	–	CellTrace™ Violet	–	Invitrogen	–

While the CD3, CD4, CD8α, TCR-γδ, CRR7, and Live/Dead staining was included in both panels, the IFN-γ staining (*) was only included in the IFN-γ analysis (Figure 7) and the proliferation (#) staining only in the proliferation analysis (Figure 6).

### The porcine reproductive and respiratory syndrome virus challenge-strain specific IFN-γ production of T cells

PBMCs were plated at 500,000 cells/well and allowed to rest overnight. The following day, cells were stimulated with either media (MOCK), NC174, NADC20, NADC30, or VR2332 (MOI of 0.1); Phorbol 12-myristate 13-acetate (PMA, 5 ng/mL, Alfa Aesar, Ward Hill, MA, USA)/Ionomycin (500 ng/mL, AdipoGen, San Diego, CA, USA) was used as a positive control. Plates were cultured for 18 h; Monensin (5 μg/mL, Alfa Aesar) was added for the last 4 h of culture. Eight replicates were then pooled and stained for flow cytometry analysis according to Table 1. Data were acquired on a Cytoflex using the CytExpert software (Beckman Coulter). Data analysis was performed with FlowJo version 10.5.3 (FLOWJO LLC) with gates based upon the FMO controls.

### Statistical analysis

Statistical analysis was performed using GraphPad Prism 9.1.1 (GraphPad Software, San Diego, CA, USA). All qPCR data were log-transformed prior to statistical analysis. Depending on the dataset, statistical significance was analyzed by either two-way ANOVA or a two-tailed unpaired Student *t*-test. Multiple comparisons were performed using either Tukey’s or Šidák multiple comparisons test.

## Results

### Heterologous vaccine efficacy

**FIGURE 2:**
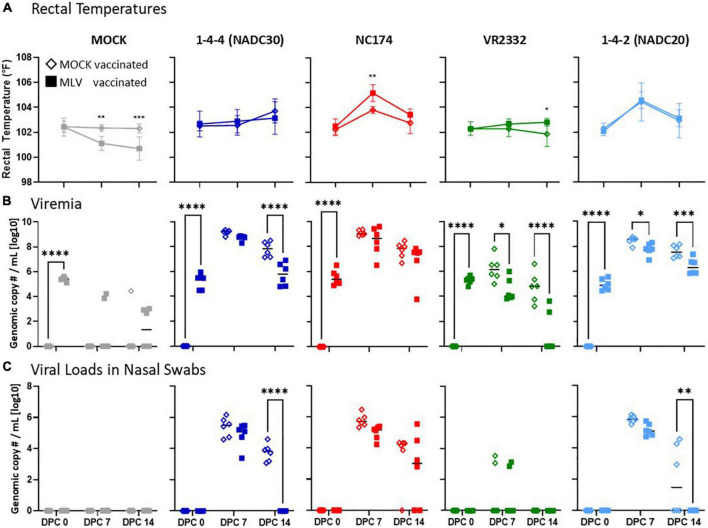
Heterologous vaccine efficacy of Prevacent—rectal temperatures, viremia, and viral loads in nasal swabs. Rectal temperatures **(A)**, viremia **(B)**, and **(C)** viral loads in nasal swabs were determined at 0, 7, and 14 days post challenge (dpc) with MOCK (gray), or the PRRSV strains 1–4–4 (NADC30, dark blue), NC174 (red), VR2332 (green), or 1–4–2 (NADC20, light blue). The line graphs in panel **(A)** illustrate the means with standard deviation of rectal temperatures [°C]. Viremia **(B)** and viral shedding **(C)** were quantified by PRRSV-specific qPCR in serum and nasal swabs, respectively (genomic copy numbers/mL [log10]). The black bars represent the median values; in addition, individual data points are shown for MOCK vaccinated animals (open diamonds) and MLV vaccinated animals (filled squares). The data were analyzed using Šidák multiple comparison test 2-way ANOVA. Each vaccinated group was compared to their respective PRRSV type-2 challenge MOCK vaccinated group within each timepoint. ^****^p < 0.0001, ^***^p < 0.001, ^**^p < 0.01, *p < 0.05.

**FIGURE 3:**
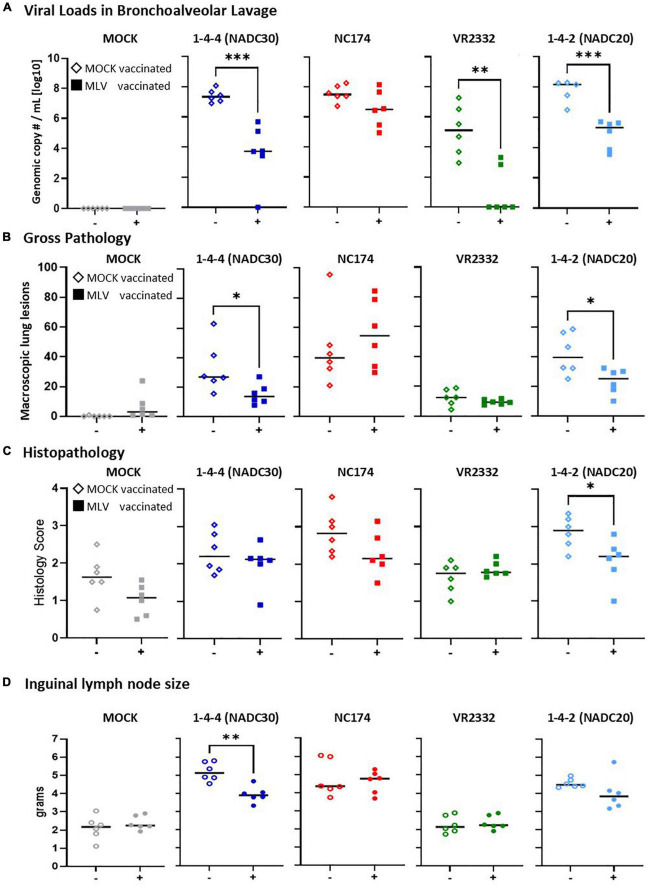
Heterologous vaccine efficacy—lung viral loads, pathology, and inguinal lymph nodes size. **(A)** Viral loads in lung were assessed in bronchoalveolar lavage (BAL) by PRRSV-specific qPCR (genomic copy numbers/mL [log10]). Of note, while PRRSV was not detected in BAL of one MOCK-vaccinated/NADC20-challenged animal, this pig had the second highest lung pathology score combined with the highest PRRSV load in the 14 dpc nasal swab. Based on this discrepancy, it was excluded from this analysis. **(B,C)** Lung gross- and histopathology of all seven lobes were assessed by a blinded veterinarian at 14 days post challenge (dpc). Panel **(B)** depicts the percental lung lesions for each individual pig. Panel **(C)** shows the histopathology scores of all seven lobes following the scoring guidelines of Halbur et al. (1995). Each vaccinated group was compared to their respected PRRSV type-2 challenge unvaccinated group using a two-tailed unpaired *t*-test. The black bars represent the median values; in addition, individual data points are shown for MOCK vaccinated animals (open diamonds) and MLV vaccinated animals (filled squares). **(D)** The superficial ingual lymph nodes were collected and weighted. The average weight from the left and right lymph nodes is shown for each pig. Each vaccinated group was compared to their respective PRRSV type-2 challenge unvaccinated group. Data comparison was performed using a two-tailed unpaired *t*-test. ***p < 0.001, **p < 0.01, *p < 0.05.

The heterologous vaccine efficacy of Prevacent was determined in three ways: (i) clinical signs including rectal temperatures, (ii) PRRSV loads in nasal swabs and serum were assessed at 0, 7, and 14 dpc (Figure 2); and (iii) viral loads, and the lungs were macroscopically assessed at necropsy and sent for microscopic examination (14 dpc, Figure 3).

#### Weight gains, clinical signs, and viral loads in serum, BAL, and nasal swabs

There were no relevant differences in weight gains between the groups. Clinical signs were mild; they included lethargy and respiratory distress at around 7–14 dpc. Only two differences between MOCK- and Prevacent-VAC groups were noticed: (i) Within NC174-CHA groups, the Prevacent-VAC pigs were slightly less lethargic; and (ii) in contrast to the Prevacent-VAC pigs, the MOCK group exhibited pronounced clinical signs upon challenge with NADC20: MOCK-pigs showed signs of respiratory distress, strong lethargy, and anorexia from 7 to 14 dpc (d.n.s.). Rectal temperatures varied between groups (Figure 2A): only the NC174- and NADC20-CHA pigs exhibited increased temperatures at 7 dpc. Within NC174-CHA groups, the Prevacent-VAC pigs had at that time point even a higher temperature compared to their MOCK-VAC pigs. Body temperatures upon challenge with NADC20 were similar between the MOCK- and Prevacent-VAC pigs. Taken together, under the present research conditions, clinical signs were mostly mild and included lethargy, anorexia, respiratory disease, and some elevated temperatures for NC174 and NADC20. The most prominent protective aspect of Prevacent were the reduced respiratory disease, anorexia, and lethargy after NADC20 challenge.

With the limited clinical signs, viral load quantification in nasal swabs and sera were performed to better evaluate the heterologous vaccine efficacy (Figures 2B,C). Pre-challenge viral load analysis at 0 dpc showed that all Prevacent-VAC animals had similar PRRSV viral copy numbers: this confirms that Prevacent vaccination was not only successful but also homogenous (Figure 2B). Challenge with the different PRRSV strains induced viremia that peaked at 7 dpc. At that time, challenge with VR2332 led to a mild to moderate viremia with a median genomic copy number of 10^6.2 in the MOCK-VAC groups. In contrast, NC174, NADC30, and NADC20 challenge induced strong viremia in MOCK-VAC pigs: median genomic copy numbers/mL were 10^9.0, 10^8.6, and 10^9.2, respectively. At 14 dpc, viremia decreased by ∼1–2 logs. For VR2332 and NADC30, Prevacent vaccination significantly reduced viremia at both time points; for NADC30, it reduced viremia at 14 dpc (Figure 2B). Of note, while all MOCK-VAC pigs remained viremic upon VR2332 challenge, 4/6 Prevacent-VAC pigs could clear this PRRSV strain by 14 dpc.

In addition to the PRRSV-generic qPCR, a Prevacent-specific qPCR analysis has been performed for sera at 7 and 14 dpc: this goal of this analysis was to provide insight into the contribution of the Prevacent vaccine strain and the challenge strains to the overall PRRSV load in sera. This analysis showed that (i) at 14 dpc, Prevacent was not detected at 14 dpc (d.n.s); and (ii) at 7 dpc, it was either cleared from sera or present at only very low levels (Ct ≥ 31). Interestingly, while most animals (4/6) in the MOCK- and VR2332-CHA groups still had detectable levels of the Prevacent vaccine strain in sera, all or 5/6 animals within the NADC30, NC174, and NADC20 groups cleared the Prevacent vaccine strain. These data show that pigs challenged with PRRSV strains that induce high viremia cleared the Prevacent strain faster (Supplementary Figure 1 and d.n.s.).

Pig viral loads were also assessed in nasal swabs to evaluate viral shedding. Importantly, the Prevacent vaccine strain was not detected in nasal swabs at the analyzed time points—0, 7, and 14 dpc (=28, 35, and 42 dpv, respectively; d.n.s.). In VR2332-CHA pigs, viral loads in nasal swabs were either low (<10^4^ genomic copy number/ml in 4/12 pigs) or completely absent (8/12 pigs; Figure 2C). In contrast, challenge with the other PRRSV strains led to considerably higher viral loads in nasal swabs of all inoculated animals (include mean genomic copy number range; Figure 2C). As viremia, viral loads in nasal swabs peaked at 7 dpc. Prevacent vaccination led by number to a decrease in viral loads in nasal swabs at 7 dpc for NC174, NADC20, and NADC30. At 14 dpc, Prevacent vaccination both significantly reduced and completely cleared the viral loads in nasal swabs of NADC20- and NADC30-CHA pigs.

At necropsy, viral loads were additionally quantified *via* universal PRRSV-specific qPCR in BAL (Figure 3A). All MOCK-CHA pigs were PRRSV-2 negative in BAL. Within the NADC30, NC174, and NADC20 challenge groups, MOCK-VAC pigs showed with ∼10^8^ genomic copy numbers per mL BAL the highest median viral loads. Prevacent vaccination could drop these viral loads by number to 10^6.5 for NC174, and significantly to 10^3.7 for NADC30, 10^5.3 for NADC20. While all MOCK-VAC pigs within the VR2332-CHA groups had detectable PRRSV levels between 10^3 and 10^8, 4/6 Prevacent-VAC pigs cleared PRRSV from BAL.

In conclusion, Prevacent was absent in nasal swabs at 4–6 weeks post vaccination; and it was either absent or present at low levels in sera at these time points. Regarding heterologous vaccine efficacy, Prevacent vaccination significantly reduced viremia upon VR2332 challenge; it limited both viremia and viral shedding in NADC20- and NADC30-CHA groups; and Prevacent significantly reduced the BAL viral loads of NADC20, NADC30, and VR2332 (Figure 3A).

#### Vaccine efficacy in tissues—viral loads, lung pathology, and lymph node sizes

At necropsy, viral loads were assessed *via* PRRSV-specific qPCR in BAL (Figure 3A). All MOCK-CHA animals were negative. MOCK-VAC animals from the NADC30, NC174, and NADC20 groups had viral loads of ∼10^8^ genomic copy numbers/mL. In contrast, the MOCK-inoculated and VR2332-CHA animals had with ∼10^5^ a roughly 1,000x fold lower median viral load. Prevacent vaccination could reduce by number the median viral loads of all challenge strains. This reduction became significant for NADC30, VR2332, and NADC20. Of note, the BAL from 4/6 Prevacent-VAC and VR2332-CHA animals were PRRSV-2 negative.

Pulmonary gross changes were mainly absent in both MOCK-CHA groups and minimal in VR2332-CHA groups; yet they were clearly present in NC174-, NADC20-, and NADC30-CHA pigs (Figure 3B). Prevacent vaccination reduced the lung gross lesions in pigs challenged with two of the three pathology-inducing strains—NADC30 and NADC20. As seen in the macroscopic findings, median histopathological changes in MOCK-VAC pigs were also highest in the NADC30, NC174, and NADC20 groups. However, histopathological changes were also present in the MOCK- and VR2332-CHA groups (Figure 3C). Comparing MOCK and Prevacent-VAC groups, the histopathology analysis revealed only one difference: Within the NADC20-CHA groups, Prevacent-VAC animals had significantly lower histopathology scores compared to MOCK-VAC animals.

Along with lung pathology, inguinal lymph nodes were assessed for weight as an increase in lymph node size is often associated with inflammation. Both MOCK and VR2332 groups remained around about a healthy weight throughout the study (median weigh ∼2 g). PRRSV-2 challenge caused the lymph nodes to enlarge for NC174, NADC20 and, NADC30. Prevacent was able to significantly reduce the lymph node size for NADC30 by 1 g (4.7–3.6 g median weight).

Conclusively, Prevacent reduced the BAL viral loads for NADC30, VR2332, and NADC20. While VR2332 only induced minimal lung lesions, Prevacent did reduce not only the lung gross- and/or histopathology lesion scores but also the median inguinal lymph node weights for NADC30 and (by number) for NADC20.

### Heterologous vaccine immunogenicity

**FIGURE 4:**
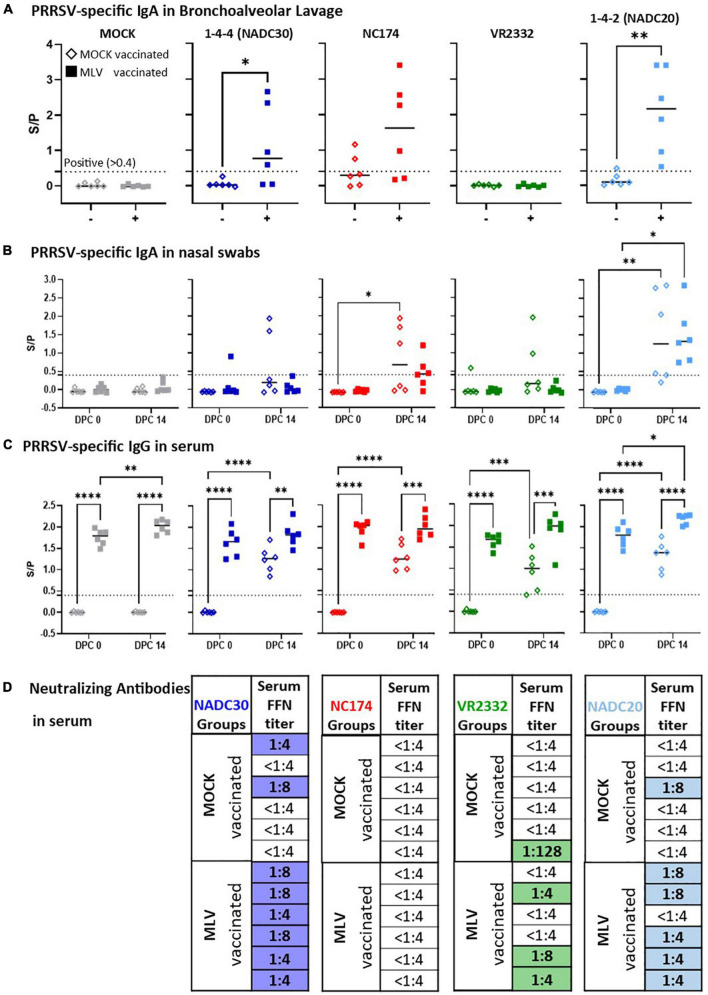
Heterologous vaccine immunogenicity—the humoral immune response. Immunoglobulin A of **(A)** bronchoalveolar lavage (BAL), **(B)** nasal swabs, and **(C)** serum IgG levels were evaluated at 0 and 14 days post challenge (dpc) *via* a PRRSV X3 ELISA. The IgA and IgG ELISA S/P ratios were compared within their challenge groups—MOCK (gray), 1-4-4 (NADC30, dark blue), NC174 (red), VR2332 (green), and 1-4-2 (NADC20, light blue). The black bars represent the median values; in addition, individual data points are shown for MOCK vaccinated animals (open diamonds) and MLV vaccinated animals (filled squares). Data were statistically analyzed using a 2-way ANOVA with time and vaccination as the two parameters and Tukey’s multiple comparison test. ^****^p < 0.0001, ^***^p < 0.001, ^**^p < 0.01, *p < 0.05. **(D)** Neutralizing antibody (NA) titers determined *via* FFN test against the respective challenge strain at 0 and 14 dpc. Since no animals showed FFN titers at 0 dpc, only the 14 dpc data are shown. Titers ≥ 1:4 were considered positive. The titer for each individual PRRSV-challenged pig is shown. Positive NA titers are highlighted in blue (NADC30), red (NC174, not detected), green (VR2332), and light blue (NADC20).

**FIGURE 5:**
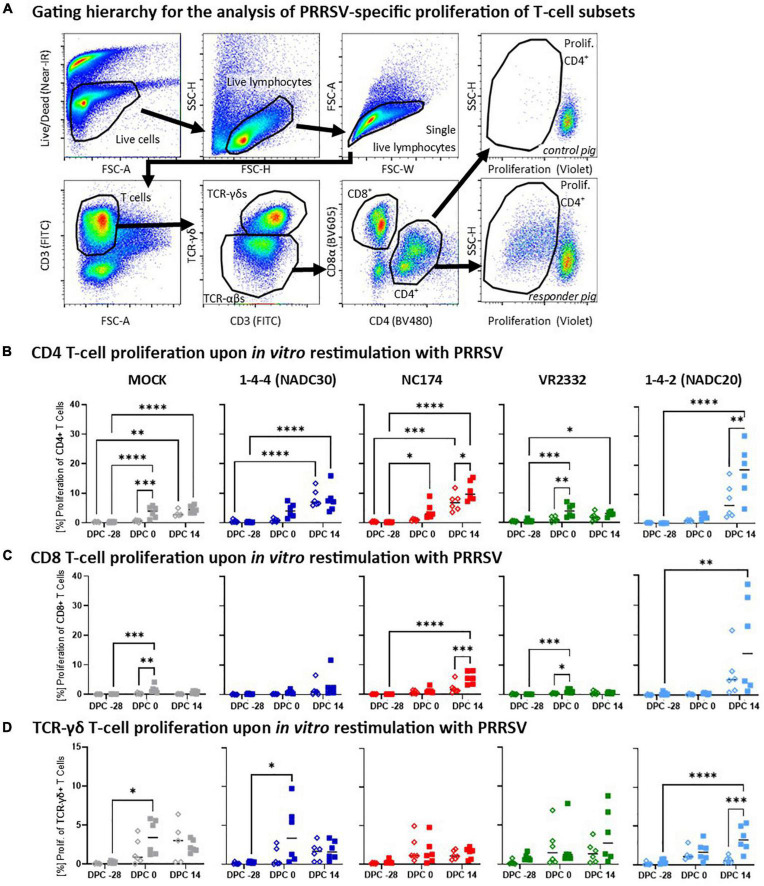
Heterologous vaccine immunogenicity—proliferation of CD4, CD8, and TCR-γδ T cells. Panel **(A)** shows the gating hierarchy to assess the heterologous proliferative response of T-cell subsets to the respective PRRSV type-2 challenge strains. A live/dead discrimination dye was included to exclude dead cells. Live cells were used to identify live lymphocytes *via* a FSC/SSC lymphocyte gate. From live lymphocytes, doublets were excluded using a FSC-width (FSC-W)/FSC-area (FSC-A) gate on singlets. These single living lymphocytes were used to gate on T cells (FSC-A/CD3), and further to discriminate TCR-αβ and TCR-γδ T cells. TCR-αβ T cells were further divided into CD4 and CD8 T cells *via* their CD4/CD8α expression profile. Proliferation of the CD4, CD8, and TCR-γδ T cells was identified *via* a violet proliferation dye. Two examples demonstrate representative staining patterns of a control animal (top right plot) and a high responder animals (bottom right plot). Panels **(B–D)** show the proliferative responses of CD4 **(B)**, CD8 **(C)**, and TCR-γδ T cells **(D)** according to their challenge groups—MOCK (gray), NC174 (red), NADC20 (light blue), NADC30 (dark blue), and VR2332 (green). The black bars represent the median values; in addition, individual data points are shown for MOCK vaccinated animals (open diamonds) and MLV vaccinated animals (filled squares). Data were statistically analyzed using a 2-way ANOVA with time and vaccination as the two parameters and Tukey’s multiple comparison test. ^****^p < 0.0001, ^***^p < 0.001, ^**^p < 0.01, *p < 0.05.

**FIGURE 6:**
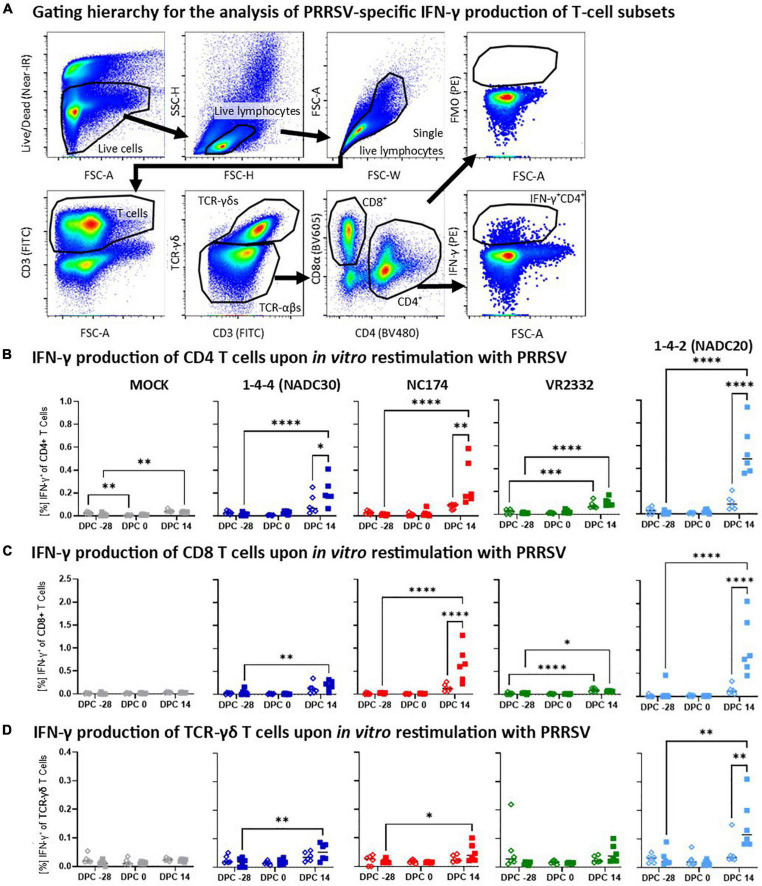
Heterologous vaccine immunogenicity—IFN-γ production of CD4, CD8, and TCR-γδ T cells. **(A)** Gating hierarchy to assess the heterologous IFN-γ response of T-cell subsets to the respective PRRSV type-2 challenge strains. The gating hierarchy follows largely the proliferation analysis shown in Figure 5. However, instead of gating on proliferating cells, IFN-γ was analyzed in a FSC-A/IFN-γ plot. The IFN-γ gate was set using the appropriate FMO control (top right plot). Panels **(B–D)** show the IFN-γ responses of CD4 **(B)**, CD8 **(C)**, and TCR-γδ T cells **(D)** according to their challenge groups—MOCK (gray), 1-4-4 (NADC30, dark blue), NC174 (red), VR2332 (green), and 1-4-2 (NADC20, light blue). The black bars represent the median values; in addition, individual data points are shown for MOCK vaccinated animals (open diamonds) and MLV vaccinated animals (filled squares). Data were statistically analyzed using a 2-way ANOVA with time and vaccination as the two parameters and Tukey’s multiple comparison test. ^****^p < 0.0001, ^***^p < 0.001, ^**^p < 0.01, *p < 0.05.

**FIGURE 7:**
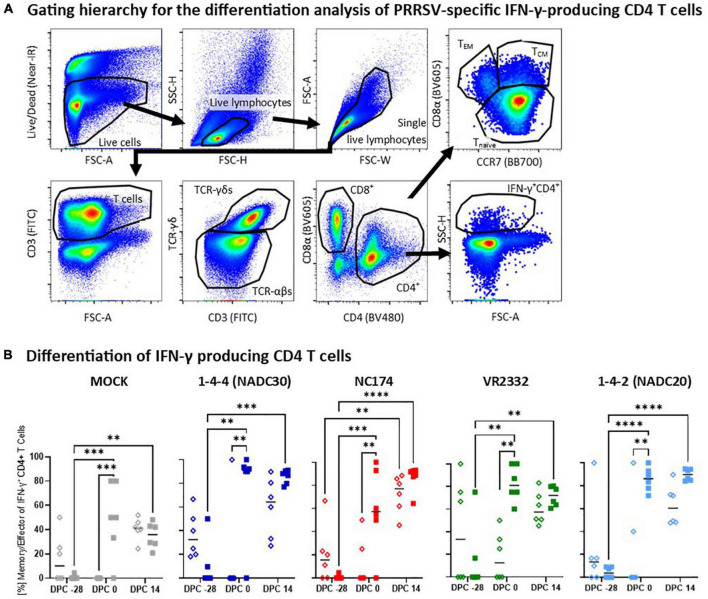
Heterologous vaccine immunogenicity—differentiation of IFN-γ producing CD4 T cells. Panel **(A)** shows the gating hierarchy to assess the differentiation of IFN-γ producing CD4 T cells. After gating on IFN-γ + CD4 T cells as described in Figure 6, their differentiation was analyzed *via* their CD4/CD8α expression profile to distinguish naïve (CCR7^+^CD8α^–^), central memory (T_CM_, CCR7^+^CD8α^+^) and effector memory (T_EM_, CCR7^–^CD8α^+^) CD4 T cells (top right plot). Since the vast majority of CD8α^+^ IFN-γ-producing CD4 T cells belonged to the T_CM_ subset (d.n.s.), both T_CM_ and T_EM_ were combined in the downstream analysis into the “memory/effector” subset. Panel **(B)** shows the frequency of these memory/effector within IFN-γ-producing CD4 T cells according to their challenge groups—MOCK (gray), 1-4-4 (NADC30, dark blue), NC174 (red), VR2332 (green), and 1-4-2 (NADC20, light blue). The black bars represent the median values; in addition, individual data points are shown for MOCK vaccinated animals (open diamonds) and MLV vaccinated animals (filled squares). Data were statistically analyzed using a 2-way ANOVA with time and vaccination as the two parameters and Tukey’s multiple comparison test. ^****^p < 0.0001, ^***^p < 0.001, ^**^p < 0.01.

In conjunction with vaccine efficacy, heterologous vaccine immunogenicity was investigated—both the humoral and T-cell immune response. The humoral immune response was studied by quantifying the local anti-PRRSV IgA levels in nasal swabs and BAL, and the serum anti-PRRSV IgG and nAb levels (Figure 4). To study the T-cell response, PBMC were isolated, *in vitro* restimulated with the respective PRRSV challenge strains, and analyzed *via* polychromatic flow cytometry for three main readout parameters—(i) proliferation (Figure 5) and (ii) IFN-γ production (Figure 6) of CD4, CD8, and TCR-γδ T cells, and (iii) CD4 T-cell differentiation into memory/effector cells (Figure 7).

#### Humoral immune response

The local humoral immune response was studied by quantifying anti-PRRSV IgA levels in BAL at necropsy (Figure 4A) and in nasal swabs at 0 and 14 dpc (Figure 4B). Of note, in contrast to the non-diluted nasal swab samples, the BAL samples were diluted 1:200 before analysis. At 2 weeks post challenge, the majority of BAL samples from MOCK-VAC pigs were negative for PRRSV-specific IgA. While the BAL from vaccinated and VR2332-CHA pigs were also negative, Prevacent vaccination induced a strong IgA response for NADC30, NC174, and NADC20: 4/6 of the Prevacent-VAC pigs in the NADC30 and NC174 pigs and all Prevacent-VAC pigs in the NADC20 challenge group had S/P ratios of >0.4. Thereby, Prevacent increased the lung PRRSV-specific IgA levels for NC174 (by number) and significantly for NADC30 and NADC20.

The IgA levels in nasal swabs were considerably lower (Figure 3B): except for some outliers, IgA levels in the MOCK-, NADC30- and VR2332-CHA pigs remained below an S/P ratio of 0.4. However, challenge with NC174 and NADC20 induced an observable and mostly significant local IgA response. Yet, there was no difference between the respective MOCK- and Prevacent-VAC groups.

The systemic humoral immune response was evaluated in two ways—anti-PRRSV IgG and challenge-strain specific nAb levels in serum (Figures 4C,D). Four weeks post vaccination, so at 0 dpc, every vaccinated animal but no control animal had a high positive IgG level—S/P 1.3—2.1. By 14 dpc, infection with each of the four PRRSV strains also induced anti-PRRSV serum IgG in MOCK-VAC animals; yet all vaccinated animals had significantly higher serum IgG levels than their respective MOCK-VAC groups (Figure 4C). The challenge-strain specific serum nAb titers were determined by an FFN test at 0 and 14 dpc (Figure 4D). No nAbs were detected at 0 dpc (d.n.s). At 14 dpc, neither the MOCK- (d.n.s.) nor the NC174-CHA groups developed nAbs against the challenge strain either. However, NADC20, NADC30 and VR2332 challenge induced mainly low-titer serum nAbs by 14 dpc: out of the six pigs per group, only 1–2 pigs developed serum nAb titers in the MOCK-VAC animals; in contrast, 3/6, 5/6, and 6/6 pigs in the Prevacent-VAC groups developed nAb against the VR2332, NADC20, and NADC30 challenge strains, respectively (Figure 4D).

These data demonstrate that Prevacent vaccination induced a strong local IgA response in BAL for NADC30, NC174 (by number), and NADC20; it also induced a systemic humoral immune response with high serum IgG titers in all groups and a higher post-challenge frequency of nAb positive animals against VR2332, NADC30, and NADC20.

#### Heterologous vaccine immunogenicity—proliferation of T-cell subsets

Along with the humoral response, the cellular immune response is crucial for the protection against PRRSV. To provide a more detailed understanding of the MLV-induced heterologous vaccine immunogenicity, we investigated the PRRSV-strain specific proliferative (Figure 5) and IFN-γ (Figure 6) response of CD4, CD8, and TCR-γδ T cells.

The proliferative response of CD4, CD8, and TCR-γδ T cells was analyzed after *in vitro* restimulation with the respective challenge PRRSV-2 strains (MOI 0.1) by multi-color flow cytometry—gating hierarchy shown in Figure 5A. At −28 dpc, CD4 T cells showed a very limited background proliferation (Figure 5B). Four weeks later (0 dpc) and in contrast to MOCK-VAC pigs, CD4 T cells from Prevacent-VAC pigs started to develop a heterologous proliferative response. This response was moderate for NADC20, clearly visible for NADC30 and NC174, and significantly for the MOCK- and VR2332-CHA groups. At 14 dpc, the proliferative CD4 T-cell response was significantly increased also in Prevacent-VAC animals of the NC174 and NADC20 groups. In contrast to CD4 T cells, CD8 T cells showed mostly a lower proliferative response (Figure 5C). Yet, they displayed a similar pattern: (i) generally, the proliferative response was increasing over time; (ii) at 0 dpc, mainly the MOCK and VR2332 groups experienced an increased proliferation; and (iii) post-challenge, Prevacent could boost their proliferation in the NC174 group. The proliferative TCR-γδ response showed a higher within-group variability. Comparing MOCK- and Prevacent groups, the only clear and significant effect of Prevacent was an increase in TCR-γδ at 14 dpc against the NADC20 strain (Figure 5D). Collectively, these data indicate that while the effect on TCR-γδ proliferation was limited to NADC20, Prevacent vaccination increased the proliferation of CD4 and CD8 T cells against three heterologous PRRSV-2 strains—VR2332 (pre-challenge), NC174 (post-challenge), and NADC20 (CD4 T cells only, post-challenge).

#### Heterologous vaccine immunogenicity—IFN-γ production of T-cell subsets

In addition to the systemic proliferative response, heterologous vaccine immunogenicity was also evaluated by studying the arguably most relevant antiviral T-cell cytokine—IFN-γ (Figure 6). The gating hierarchy used to selectively analyze the IFN-γ production of CD4, CD8, and TCR-γδ T cells is similar to the proliferation analysis and shown in Figure 6A. Pre-challenge (−28 and 0 dpc), IFN-γ production was low in all T-cell subsets with no significant differences between the respective vaccination groups (Figures 6B–D). In contrast, at 14 dpc, there was a notable IFN-γ production in most PRRSV-2-CHA groups. For CD4 T cells, this post-challenge response was significantly increased by Prevacent vaccination against NC174, NADC20, and NADC30 (Figure 6B). In CD8 T cells, Prevacent significantly boosted the IFN-γ response against NC174 and NADC20 (Figure 6C); and for TCR-γδ T cells, Prevacent vaccination led to an increased IFN-γ production against NADC20 (Figure 6D). Therefore, Prevacent vaccination could boost the heterologous post-challenge IFN-γ response against NADC30 (in CD4 T cells), NC174 (in CD4 and CD8 T cells), and NADC20 (in all T-cell subsets).

#### Heterologous vaccine immunogenicity—differentiation of IFN-γ producing CD4 T cells

While the heterologous IFN-γ production showed the strongest response post-challenge, T-cell differentiation analysis of the IFN-γ producing T cells revealed remarkable pre-challenge differences (Figure 7). Once more, a multi-color flow cytometry with a sophisticated gating hierarchy was used to assess the differentiation of IFN-γ producing CD4 T cells into CCR7^+^CD8α^–^ naïve, CCR7^+^CD8α^+^ central memory (T_CM_) and CCR7^–^, CD8α^+^ effector memory (T_EM_) CD4 T cells (Figure 7A). Since the vast majority of IFN-γ producing CD8α^+^ CD4 T cells belonged to the CCR7^+^ T_CM_ subset (d.n.s.), the antigen-experienced T_CM_ and T_EM_ subsets were combined into one “memory/effector” subset (Figure 7B). Pre-vaccination, so at −28 dpc, the majority of IFN-γ was produced by naïve CD4 T cells—median: 10–40% memory/effector CD4 T cells. At 0 dpc, IFN-γ in the MOCK-VAC groups was still mainly produced by naïve CD4 T cells—median 0-15% memory/effector CD4 T cells. In contrast, in Prevacent-VAC groups, IFN-γ was mainly produced by memory/effector CD4 T cells—median ∼50% to >90%. This difference in pre-challenge differentiation of IFN-γ producing CD4 T cells was significant for all groups and PRRSV-2 challenge strains. At 14 dpc, the frequency of memory/effector CD4 T cells increased in the MOCK-VAC groups; yet, at least by number, the Prevacent-VAC groups still had a higher median frequency of memory/effector cells within all PRRSV-2-CHA groups—Figure 7B. Conclusively, this CD4 differentiation analysis reveals important immune mechanisms in heterologous vaccine immunogenicity: while Prevacent increased the CD4 IFN-γ response not before challenge, it already promoted the pre-challenge differentiation of these CD4 T cells against every analyzed PRRSV-2 strain—NC174, NADC20, NADC30, and VR2332.

### Immune correlates of protection

The data above demonstrate that Prevacent showed various degrees of heterologous vaccine immunogenicity and efficacy. An important parameter barely analyzed for PRRSV are immune correlates of protection (CoP) (Plotkin and Gilbert, 2012). These correlates can facilitate vaccine development as well as forecasting of vaccine efficacy against emerging PRRSV strains. To provide insight into potential CoPs for heterologous PRRSV strains, we performed correlation analysis between the analyzed pre-challenge immune parameters (0 dpc) and three post-challenge (14 dpc) parameters associated with protection—lung pathology, viral shedding, and viremia (Table 2). Negative correlations (*R* = 0 > −1) indicate that an increase in the pre-challenge immune parameter correlates with a reduced pathology or viral load. Neither the systemic CD8 and TCR-γδ response correlated well with protection: only CD8 proliferation negatively correlated with NADC30 shedding; the TCR-γδ IFN-γ response even significantly correlated positive with NADC20 shedding. In contrast, with the exception of NC174 gross pathology, the CD4 T-cell response correlated negatively with all analyzed parameters of protection. The strongest and most significant CD4 correlations were observed for the NADC20 and/or NADC30 strains: the CD4 IFN-γ response and differentiation into memory/effector cells significantly correlated negative with NADC20-induced lung gross pathology; CD4 T-cell proliferation showed a significant negative correlation with NADC20 shedding and viremia; and all CD4 parameters (proliferation, IFN-γ, and differentiation) correlated with both NADC30 shedding and viremia. Regarding the humoral immune response, while IgA levels in nasal swabs (local IgA) showed both positive and negative correlations with protection, the systemic IgG levels correlated well with most protection parameters: Systemic IgG level significantly correlated negative with NADC20-induced gross pathology, NADC30-induced shedding, and with viremia induced by NADC30, VR2332, and NADC20. These data demonstrate the while only the T-cell response was analyzed in a strain-dependent manner, both systemic IgG levels and the CD4 T-cell response are candidates to serve as important CoP for PRRSV.

**TABLE 2:** Immune correlates of protection.

Protection		Gross pathology				Shedding				Viremia				-1
**Response (0 dpc)**		**NADC30**	**NC174**	**VR2332**	**NADC20**	**NADC30**	**NC174**	**VR2332**	**NADC20**	**NADC30**	**NC174**	**VR2332**	**NADC20**	**0.9** **0.8**

TCR-γδs	Proliferation	*−0.37*	*−0.27*	*0.01*	*−0.42*	*−0.50*	*0.02*		*−0.19*	*−0.42*	*0.29*	*−0.17*	*−0.49*	**0.7**
														**0.6**
	IFN-γ	*−0.23*	*0.28*	*−0.26*	*0.46*	*−0.19*	*0.09*	**n.d**	**0.72**	*0.04*	*−0.11*	*0.39*	*0.36*	**0.5** **0.4**

CD8	Proliferation	*−0.37*	*0.34*	*−0.22*	*−0.09*	−**0.61**	*0.20*		*−0.35*	*−0.45*	*0.11*	*−0.41*	*−0.31*	**0.3** **0.2**
	IFN-γ	*0.26*	*0.54*	*0.46*	*0.11*	*0.27*	*−0.18*	**n.d**	*0.01*	*−0.04*	*−0.23*	*−0.22*	*0.21*	**0.1**
														**0**

CD4	Proliferation	*−0.46*	*0.22*	*−0.36*	*−0.48*	−**0.72**	*−0.40*		−**0.56**	−**0.64**	*−0.22*	*−0.57*	−**0.73**	**0.1** **0.2**
	IFN-γ	*−0.36*	*−0.19*	*−0.32*	−**0.59**	−**0.75**	*−0.43*	**n.d**	*−0.49*	−**0.58**	*−0.17*	*−0.34*	*−0.52*	**0.3** **0.4**
	Memory	*0.02*	*0.29*	*−0.13*	−**0.77**	−**0.61**	*−0.16*		*−0.40*	−**0.70**	*−0.36*	*−0.59*	−**0.74**	**0.5** **0.6**

Humoral	Local IgA	*−0.28*	*0.24*	*−0.21*	*−0.45*	*−0.35*	*−0.01*		*−0.54*	*−0.15*	*−0.36*	*0.17*	−**0.60**	**0.7** **0.8**
	Systemic IgG	*−0.55*	*0.23*	*−0.37*	−**0.59**	−**0.95**	*−0.18*	**n.d**	*−0.55*	−**0.73**	*−0.27*	−**0.86**	−**0.78**	**0.9** **1**

The table lists the R values for the correlations between various immune parameters at 0 dpc (e.g., proliferation, IFN-γ production, and differentiation into CD4 memory cells) and the three protective measures (gross pathology, shedding, and viremia) at 14 dpc. While numbers in italics represent non-significant correlations, the bold numbers emphasize significant correlations (p < 0.05).

## Discussion

This study sought to assess the broad protection and complex role of the local and systemic, as well as the humoral and T-cell immune system induced by the vaccine Prevacent—an MLV vaccine derived from a PRRSV-2 lineage 1 strain. While many PRRSV MLV vaccines offer high or complete protection against homologous strains, few offer similar levels of protection against heterologous strains (Diaz et al., 2012; Chae, 2021). Therefore, the overall objective of this study was to evaluate the vaccine efficacy and immunogenicity against different heterologous PRRSV-2 strains. This study investigated the effect of Prevacent on local and systemic viral loads, clinical lung lesions, as well as the humoral and T-cell mediated immune response against four PRRSV-2 strains—two lineage 1 strains [NC174 (lineage 1A) and NADC30 (lineage 1C)], one lineage 5.1 strain [VR2332], and one lineage 8 strain [NADC20]. ORF5 sequencing, performed by ISU VDL, determined that Prevacent shared the highest homology with NC174 and NADC30 (89.6%), then NADC20 (87.4%), and lastly, VR2332 (87.1%). As vaccines tend to offer better protection toward more similar strains, we expected that the highest protection would be seen against the groups challenged with the lineage 1 strains (Geldhof et al., 2012; Li et al., 2014). However, this was not necessarily the case in this study. Previous studies also noted a lack of heterologous immunogenicity and/or protection to strains with similar ORF5 sequences (Prieto et al., 2008; Diaz et al., 2012; Savard et al., 2016; Kick et al., 2019, 2021a,b). This finding supports a finding from Prieto et al. (2008) that indicates that ORF5 sequencing may not be the best indicator to predict vaccine-induced heterologous protection. Since ORF5 sequencing alone seems to be a weak indicator of heterologous vaccine immunogenicity and efficacy, we furthermore studied if the analyzed parameters could serve as CoPs. These CoPs can be powerful tools to guide vaccine development as well as the usage of commercially available PRRSV MLV vaccines. Thereby, this study not only aimed at providing information on heterologous immunogenicity and efficacy of the PRRSV MLV vaccine Prevacent but it also used the generated data to facilitate future vaccine development and PRRSV management.

Prevacent vaccination successfully induced viremia at 4 weeks post vaccination (=0 dpc, Figure 2B). In contrast, at this pre-challenge time point, nasal swabs from all pigs were negative for PRRSV (Figure 2C). Nasal swabs from Prevacent-VAC and MOCK-CHA pigs stayed also negative until the end of the study. This demonstrates that while Prevacent vaccination was successful, the vaccine virus was not shed at any of the analyzed time points (4–6 weeks post vaccination). However, we cannot exclude vaccine shedding prior to these timepoints.

Alongside the quantifications of the PRRSV-2 viral loads using a universal qPCR approach, we also used a Prevacent-specific qPCR to quantify the prevalence of the Prevacent vaccine strain (Supplementary Figure 1). This analysis allowed us to determine if Prevacent itself was being cleared from the serum. The inability of a vaccine to clear from the blood can potentially lead to vaccine shedding: this shedding has become a significant concern of MLV vaccines (Huang and Meng, 2010; Rowland and Lunney, 2017). Furthermore, the prolonged prevalence of a PRRSV vaccine strain can lead to recombination and the emergence of new strains: in Denmark, an MLV vaccine and field strain recombined into a new virulent PRRSV-1- strain named Horsens (Kvisgaard et al., 2021). Therefore, along with offering broad protection, a safe vaccine should not only not (or minimally) be shed but it should also be cleared from the blood. While PRRSV MLV vaccines can struggle with clearing from the blood (Rowland and Lunney, 2017), those that can clear typically begin to do so around 28–36 dpv (Geldhof et al., 2012; Savard et al., 2016; Jeong et al., 2017; Madapong et al., 2020; Kvisgaard et al., 2021). Prevacent also followed this timeline: it began to clear from blood at 7 dpc (=35 dpv) in 2/3 of the vaccinated pigs; and it was undetectable in all vaccinated pigs by 14 dpc (42 dpv, Supplementary Figure 1).

Monitoring the vaccine strain through a Prevacent-specific qPCR revealed another interesting aspect between the interaction of the vaccine strain and the challenge strains: while the Prevacent vaccine strain prevailed in the majority of the MOCK-CHA pigs (4/6) or with a strain that induced low viremia (VR2332), it was cleared by 7 dpc in all or 5/6 pigs in the groups challenged with the high viremia-inducing field isolates NADC30, NC174, or NADC20 (Supplementary Figure 1). This observation indicates not only that the vaccine and challenge strains compete for susceptible host cells, but also that PRRSV-2 field strains that induce strong viremia win this competition and quickly diminish the attenuated vaccine strains.

After ensuring Prevacent vaccination was successful, vaccine efficacy was analyzed through three parameters—body temperatures (fever), lung gross and microscopic lesions, and viral loads in nasal swabs, BAL, and serum. Fever was solely induced at 7 dpc and by only two PRRSV strains—NC174 and NADC20 (Figure 2A). Prevacent did not suppress fever induced by either of those challenge strains; in contrast, it slightly increased the body temperatures in the NC174-CHA pigs. By 14 dpc, pigs in both groups overcame the fever regardless of vaccination status.

The lungs were assessed and scored macroscopically and microscopically. While the MOCK-CHA groups showed no to minimal lung gross abnormalities (Figure 2B), the median histopathology scores were between one and two. This discrepancy can be best explained by the selection criteria of the histological sample as in Halbur et al. (1995): within each lung lobe, the section with the highest lung gross abnormality was selected for histology (Halbur et al., 1995). This selection procedure can artificially exaggerate lung lesions since even lungs with as little as 1–2% of gross abnormalities can lead to elevated histopathology scores. This might have limited the readout range and sensitivity of the test. It is also plausible that even though the overall lung was within normal limits macroscopically, the tissue had already some alterations that were not severe enough to be seen or detected by the naked eye during the macroscopic examination at necropsy. Despite being a widely used method to assess lung histopathology, selecting a pre-determined region per lobe could represent an accurate alternative to determine the lung histopathology score. Nevertheless, while only a limited difference between the histopathology of MOCK control pigs and PRRSV-CHA pigs could be observed, Prevacent significantly reduced the lung histopathology lesion score in NADC20-CHA pigs (Figure 3C). Gross findings analysis showed that MOCK- and VR2332-CHA pigs showed no or only minimal gross pathological changes. Therefore, we cannot confidently conclude on the heterologous efficacy of Prevacent to protect against potential VR2332-induced lung lesions. However, since (i) Prevacent either cleared or strongly reduced VR2332 loads in BAL, and (ii) BAL viral loads will affect virus-induced lung pathology, we can assume that Prevacent has also the potential to protect against potential VR2332-induced lung lesions. In contrast to VR2332, the three PRRSV field isolates NADC30, NC174, and NADC20 induced clear gross lung changes (Figure 3B). Within these groups, Prevacent significantly reduced lung pathology induced by the lineage 1 strain NADC30 and the lineage 8 NADC20 strain.

In parallel to lung assessment and scoring, inguinal lymph nodes were extracted and weighed. Superficial inguinal lymph nodes can be used as clinical indicators of PRRSV exposure (Rossow et al., 1995). Around 7 days after infection, T cells begin to increase in number in the follicles of the lymph node. These T cells are believed to indicate CD4^+^ T cell migration into the follicle to assist in the B cell-mediated immune response; additionally, macrophages also begin to increase in the lymph nodes inducing high levels of inflammation (Garcia-Nicolas et al., 2015). Although we did not stain for cytokine expression and immune cell infiltration, we did assess the size of the inguinal lymph nodes because size has been shown to be a clinical measure for PRRSV exposure (Rossow et al., 1995). Therefore, in evaluating the inguinal lymph nodes, we wanted to determine if our challenge strains were causing the lymph nodes to enlarge and if Prevacent was able to reduce the lymph nodes in size. In our study, inguinal lymph nodes were palpated throughout the trial (d.n.s.). Both MOCK and VR2232 groups remained around a healthy weight throughout the study. At 7 dpc, inguinal lymph nodes began to enlarge for NC174, NADC20, and NADC30 (d.n.s.), and were most prominent at 14 dpc (Figure 3C). Prevacent decreased lymphadenopathy, or inflammation of the lymph nodes, for NADC20 (by number) and significantly for NADC30. This reduction coincides with the pathology of the lungs in which Prevacent reduced lung lesions for NADC20- and NADC30-CHA pigs. The enlargement in size at 7 dpc is in accordance with the literature. Healthy lymph nodes in piglets are less than 12.5 mm in size and can enlarge up to three to four times in size by 14–21 dpc (Rossow et al., 1995). In examining the lymph node data, Prevacent is protecting against the enlargement of the inguinal lymph nodes slightly against NADC20 and significantly against NADC30.

While the clinical assessments of fever, lung pathology, and lymph node size/weight have the advantage of being direct measures of the pig’s health, they also have the disadvantage of being influenced not only by PRRSV but by a plethora of other factors such as environmental factors, the pig’s health at the time of challenge, as well as other co-infections. For example, while especially the VR2332 strain did not induce fever or remarkable lung pathology, it might well have an impact on pig health under non-laboratory field conditions. Therefore, quantification of viral loads in various tissues are not only highly connected to vaccine efficacy (Wei et al., 2019), but they can also provide important additional information on the impact of vaccination on the local infection (PRRSV in BAL), systemic infection (PRRSV viremia), and viral shedding within the pig herd (PRRSV in nasal swabs). Both, viral shedding and viremia peaked as early as 7 dpc. This peak is in confirmation with other studies who have identified peak shedding and viremia to be within 3–7 dpc for both PRRSV type-2 (Fontanella et al., 2017; Kick et al., 2019, 2021a) and PRRSV type-1 strains (Bonckaert et al., 2016; Eclercy et al., 2019). For NC174, Prevacent demonstrated a mild and non-significant reduction of viral loads in nasal swabs, BAL, and viremia. In contrast, Prevacent could significantly reduce viral loads in all locations for NADC30, VR2332, and NADC20 (Figures 2B,C, 3A). Only VR2332 in nasal swabs was not significantly reduced; however, since 4/6 (7 dpc) and all pigs (14 dpc) were negative in the MOCK-VAC group, it is unlikely that Prevacent can further improve these minimal to absent VR2332 levels. Hence, except for NC174 in which the viral load reduction was rather mild and statistically non-significant, Prevacent demonstrated a strong reduction of viral loads against very diverse PRRSV-2 strains—NADC30 (lineage 1), VR2332 (lineage 5), and NADC20 (lineage 8). Prevacent can thereby reduce the effect of diverse PRRSV strains regarding lung pathology, systemic infection, and transmission.

To assess how Prevacent provided this heterologous immunity, we performed a detailed analysis of the induced adaptive immune response—both the humoral and T-cell immune response. The humoral immune response was analyzed by IgA quantification in BAL and nasal swabs as well as IgG and nAb quantification in serum. Since mucosal epithelial cells can actively transport IgA into the lumen of airways and the lung, IgA is an excellent defense mechanism to limit PRRSV infection. Additionally, local IgAs have been identified to prevent infection and limit shedding by stopping or reducing mucosal replication (Ruggeri et al., 2020). Post challenge, nasal IgA levels peaked in the presented study at 7 dpc; this result confirms the 1-week time frame to peak anti-PRRSV IgA levels in nasal swabs previously shown by Kick et al. (2021a). While the overall time frame to peak nasal IgA levels was similar between groups and animals, the quantities of IgA in nasal swabs was variable and mainly seen in NADC20-CHA pigs without a vaccine-induced effect. Within the BAL of MOCK- and VR2332-CHA groups, Prevacent likewise did not induce detectable levels of IgA. However, it has to be noted that based on the high concentration of IgA in the other groups, all nasal swab and BAL samples were analyzed at a 1:200 dilution; this means that IgA could be present in these groups at lower levels. In contrast, after challenge with a field PRRSV strain, Prevacent did boost the post-challenge BAL IgA levels: for NC174, the boost was only seen for 3/6 pigs and therefore non-significant; for NADC30 and NADC20, 4/6 and all pigs in the Prevacent-VAC groups developed considerable IgA levels. These increased BAL IgA levels in mainly the NADC30 and NADC20 groups align with the strongest protection against lung gross pathology: this finding confirms the important role of IgA in the BAL to protect the lungs against PRRSV; and it further corroborates that Prevacent shows both heterologous immunogenicity as well as protection against lung pathology induced by NADC30 and NADC20.

In contrast to the importance of lung and airway IgA in protecting pigs from infection, serum IgG and nAb levels are highly relevant in limiting viremia. Serum nAbs have even been postulated as CoPs against PRRSV (Lopez and Osorio, 2004) and reduced viremia can limit energy usage to fight off a systemic infection and thereby improve weight gains. On top, limiting viremia can also reduce the risk of PRRSV migration to other mucosal sites: this reduction can limit reproductive issues. The serum IgG levels obtained in this study provided the strongest effects of Prevacent: both, pre- and post-challenge, Prevacent significantly increased serum IgG levels in all challenge groups. However, it has to be noted that these IgG levels have not been assessed on a strain-specific basis. Therefore, we cannot conclusively determine how effective these IgGs are. If these IgGs recognize the heterologous PRRSV strains, they could limit PRRSV viremia through different mechanisms—opsonization, complement activation, and neutralization (Forthal, 2014). Opsonization promotes phagocytosis; complement activation induces both pathogen lysis and inflammation (Dimitrov and Lacroix-Desmazes, 2020). Despite the importance of these two mechanisms, their analysis is complex and is not readily available. Therefore, this study focused on the arguably most important role of antibodies—pathogen neutralization. Neutralizing antibodies have been suggested as important CoP (Lopez and Osorio, 2004). In addition, nAbs induced in pregnant sows strongly transferred to their litters; the piglets with high nAb levels also seemed to be better protected against the homologous PRRSV strain (Kick et al., 2021b). In this study, serum nAbs could be detected in 4/24 MOCK-VAC animals as early as the 14 dpc time point. This observation confirms previous reports that nAbs can be induced as early as 2 weeks post PRRSV infection (Li et al., 2014; Madapong et al., 2020; Kick et al., 2021a).

While these early nAbs appear in the minority of MOCK-VAC pigs, Prevacent-VAC animals developed serum nAbs at 14 dpc in all, 3/6, and 5/6 animals for NADC30, VR2332, and NADC20, respectively. In contrast to the previously reported data on an NC174 strain that showed broad serum nAbs at 14 dpc (Kick et al., 2021a), this NC174 strain did not induce nAbs in either group at 14 dpc. However, this NC174 strain came from a different isolate; this indicates that there can be noticeable immunogenicity differences even within different PRRSV strains. Coming back to the heterologous immunogenicity of Prevacent, this study allows two main conclusions: (i) since Prevacent only increased the systemic IgG levels but did not induce serum nAbs against NC174, the enhanced humoral immune response to this strain might be due to other antibody functions—e.g., opsonization and complement activation (Forthal, 2014); (ii) besides potential opsonizing and complement activating antibodies. While the induction of neutralizing antibodies to PRRSV has often been described to occur “delayed” after 28 weeks of vaccination/infection [reviewed in Lunney et al. (2016)], Prevacent vaccination also primed for a fast production of serum nAbs against NADC30, VR2332, and NADC20 as early as 14 dpc. Since pigs are weaned at 3–4 weeks of age and the resulting co-mingling leads to frequent PRRSV exposure, the early induction of local IgA, systemic IgG, and the increased frequency of systemic nAbs is crucial for protecting weaners against heterologous PRRSV-2 strains.

In addition to the most commonly used analysis of the humoral immune response, we included a detailed analysis of the systemic T-cell response. Blood T cells within PBMC were *in vitro* restimulated with the challenge strains: this restimulation allows the specific analysis of the heterologous T-cell response against the challenge strains. After this restimulation, polychromatic flow cytometry was used to assess both proliferation (Figure 5) as well as the IFN-γ response (Figure 6) of CD4, CD8, and TCR-γδ T cells. Concurrently, we analyzed the differentiation of IFN-γ producing CD4 T cells into memory/effector T cells (Figure 7). The analyzed parameters target the general immunology of a T-cell response: first, upon recognition of their antigen and in the presence of activation co-stimulatory signals, T cells will undergo both proliferation and differentiation. While proliferation increases the number of responding T cells, the differentiation will later provide the T cells with various effector functions such as providing B-cell help to induce a strong humoral immune response or directly limit viral propagation by the production of antiviral cytokines such as IFN-γ (Sallusto et al., 2004). In addition to improving our understanding of the underlying immunity, this detailed and strain-specific analysis of the T-cell response is crucial since T cells can provide broadly heterologous immunity (Agrawal, 2019).

Within the T-cell response, proliferation can be used to assess the general activation of T-cells. Already pre-challenge, Prevacent induced either by number or significantly an increased CD4 T-cell proliferation against all tested PRRSV strains (Figure 5B). This proliferative response increased by 14dpc: at that time point, CD4 proliferation was the strongest for the more pathogenic field strains NADC30, NC174, and NADC20. The pattern and heterogenicity of this proliferative response were comparable between CD4 and CD8 T cells; however, these CD8 T cells responded at a lower level (Figure 5C). The proliferative response of TCR-γδ T cells was limited and generally increased over time. However, the high within-group variability limits the ability to draw conclusions on a potential effect of Prevacent. The dominant role of the systemic CD4 T cells response is in line with previous studies on the T-cell response against PRRSV (Kick et al., 2019).

While Prevacent induced a proliferative response as early as 0 dpc, vaccine-induced IFN-γ production was only increased after challenge (Figure 6, 14 dpc). This is in line with the general understanding of the T-cell development and differentiation: Before acquiring an effector function like the production of IFN-γ, T-cells undergo proliferation and differentiation (Kaech et al., 2002). In accordance with this understanding, Prevacent also affected CD4 T-cell differentiation at an earlier time point: in contrast to MOCK-VAC animals, the majority of IFN-γ^+^ CD4 T cells in Prevacent-VAC animals had already differentiated into the antigen-experienced memory/effector T-cells (Figure 7). This vaccination-induced differentiation of CD4 T-cells progresses into an important post-challenge effect: at 14 dpc, the CD4 IFN-γ response is either by number (for VR2332) or significantly (for NADC30, NC174, and NADC20) increased in the Prevacent-VAC animals of all challenge groups.

In summary, Prevacent vaccination induced a broadly reactive T-cell response starting with pre-challenge T-cell proliferation and differentiation that not only can explain the strong humoral immune response and but also primed for a stronger post-challenge IFN-γ production.

In addition to studying the vaccine immunogenicity and efficacy parameters on their own, we also performed correlation analysis between the various immune and efficacy parameters to determine CoPs. This CoP analysis revealed two main CoPs—serum IgG levels and the CD4 T-cell response.

The important role of the CD4 T-cell response during viremia is supporting previous studies which identified the CD4 T cells as the major systemic T-cell responders against PRRSV (Kick et al., 2019). On the pathology side, pulmonary lesions during PRRSV infections can also be related to (often bacterial) co-infections. Probably due to the more complex mechanisms leading to lung pathology, correlation of CoP was least significant when the correlations were paired with gross lung pathology. Furthermore, correlations with viral shedding (=viral loads in nasal swabs) were not as strong as the mostly significant and strong correlations of the systemic CD4 and IgG response correlated with viremia. From a practical point of view, both immune parameters not only significantly correlated best with viremia, but all three parameters can be studied by one collection of blood into an anti-coagulant coated vacutainer. The PBMCs, once isolated, can be used to study the CD4 T-cell response and the plasma is suitable for both PRRSV and IgG quantification. Of note, while the CD4 T-cell response can be tested against specific PRRSV strains, the IgG ELISA is detecting IgGs against most, if not all PRRSV strains. Therefore, while the IgG can be suitable against some PRRSV strains with true cross-reactivity, it cannot be concluded that this analysis can be used as CoP against every PRRSV strain. Therefore, our recommendation is to use the systemic PRRSV-strain specific CD4 T-cell response as CoP since it will probably provide the most reliable correlation with heterologous protection. This important CoP can then not only facilitate PRRSV vaccine development but it can also guide the swine industry in their choice of vaccine usage: an immune biobank consisting of PBMC from pigs vaccinated with one of the available PRRSV vaccines (Chae, 2021) at the peak immune response (∼28 days post vaccination) can be restimulated with a PRRSV isolated from an emerging strain; then, the sample that shows the strongest CD4 T-cell response to this strain represents the PRRSV vaccine with the best chances for protecting against this emerging PRRSV strain.

## Summary and conclusion

This study combined Prevacent vaccination followed by *in vivo* challenge with four heterologous PRRSV strains with an extensive *ex vivo* and *in vitro* analysis of lung pathology, viral loads in various tissues, and the humoral and adaptive immune response.

The in-depth analysis of the heterologous humoral and T-cell immune response nicely explains the immunogenicity of Prevacent: early on (at 0 dpc), Prevacent induces early T-cell activation and differentiation shown by an increased proliferative response of CD8 but mainly CD4 T cells. In addition, Prevacent induced the differentiation of heterologous CD4 T cells into memory/effector cells. Downstream, this early T-cell activation and differentiation leads not only to B-cell help that drives serum IgG levels (at 0 and 14 dpc) and the frequency of nAb positive animals (14 dpc), but it also primes the vaccinated pigs for an increased post-challenge IFN-γ production (14 dpc). This induction of a combined T-cell and humoral immune response induced at least partial protection against at least three of four PRRSV strains—NADC30 (lineage 1), VR2332 (lineage 5), and NADC20 (lineage 8). The included CoP analysis revealed serum IgG levels and the CD4 T-cell response (proliferation, differentiation, and IFN-γ production) to be the best systemic CoP; however, only the CD4 T-cell response can reliably be used as CoP against specific PRRSV strains.

## Data availability statement

The original contributions presented in this study are included in the article/Supplementary material, further inquiries can be directed to the corresponding author.

## Ethics statement

The animal study was reviewed and approved by NC State University Institutional Animal Care and Use Committee (IACUC) ID# 17-166A.

## Author contributions

JP, GA, JH, and TK: conceptualization and funding acquisition. JP and TK: data curation, formal analysis, methodology, visualization, and supervision. JP, JH, and TK: project administration. JH and TK: resources. JP: writing—original draft. All authors: investigation, validation, writing—review and editing, and approve the submitted version.

## Footnotes

1
https://www.graphpad.com/quickcalcs/randomize1/

## Supplementary material

Supplementary Figure 1 Quantification of the Prevacent vaccine strain in serum: The prevalence of the Prevacent vaccine strain was quantified *via* Prevacent-specific qPCR. This table shows the Ct values of MOCK, NADC20, NC174, VR2332, and NADC20 challenged animals at 7 days post challenge. Statistical analysis has not been performed.
