# Supplementary figures and images for: Heterologous vaccine immunogenicity, efficacy, and immune correlates of protection of a modified-live virus porcine reproductive and respiratory syndrome virus vaccine
Source: Front Microbiol. 2022 Sep 23;13:977796. doi: 10.3389/fmicb.2022.977796 (PMC9537733; doi:10.3389/fmicb.2022.977796)

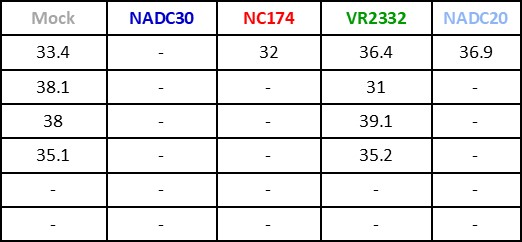

Supplement: Supplementary Figure 1 — Quantification of the Prevacent vaccine strain in serum: The prevalence of the Prevacent vaccine strain was quantified via Prevacent-specific qPCR. This table shows the Ct values of MOCK, NADC20, NC174, VR2332, and NADC20 challenged animals at 7 days post challenge. Statistical analysis has not been performed. [file Image_1.JPEG]
